# The Unmet Needs of Hepatitis E Virus Diagnosis in Suspected Drug-Induced Liver Injury in Limited Resource Setting

**DOI:** 10.3389/fmicb.2021.737486

**Published:** 2021-10-08

**Authors:** Mohamed A. El-Mokhtar, Haidi Karam-Allah Ramadan, Marwa M. Thabet, Alaa S. Abd-Elkader, Magdy Fouad, Mohammad M. Sallam, Elsayed A. Elgohary, Amer Ali Abd El-Hafeez, Mona Embarek Mohamed, Ibrahim M. Sayed

**Affiliations:** ^1^Department of Medical Microbiology and Immunology, Faculty of Medicine, Assiut University, Asyut, Egypt; ^2^Microbiology and Immunology Department, Faculty of Pharmacy, Sphinx University, Asyut, Egypt; ^3^Department of Tropical Medicine and Gastroenterology, Faculty of Medicine, Assiut University, Asyut, Egypt; ^4^Department of Clinical pathology, Faculty of Medicine, Assiut University, Asyut, Egypt; ^5^Hepato-Gastroenterology Unit, Tropical Medicine Department, Faculty of Medicine, El-Minia University, Minya, Egypt; ^6^Department of Internal Medicine, Faculty of Medicine, Zagazig University, Zagazig, Egypt; ^7^Pharmacology and Experimental Oncology Unit, Cancer Biology Department, National Cancer Institute, Cairo University, Cairo, Egypt; ^8^Department of Cellular and Molecular Medicine, University of California, San Diego, San Diego, CA, United States

**Keywords:** HEV, DILI, acute hepatitis, liver function test, screening, limited resource countries

## Abstract

**Background:** Currently, there are no specific biomarkers for drug-induced liver injury (DILI), and the diagnosis of DILI is based mainly on the exclusion of other causes of liver dysfunction and the recognition of potential causative drugs. Hepatitis E virus (HEV) diagnosis is not routinely enrolled in many countries, and HEV infection could be misdiagnosed as DILI.

**Methodology:** We retrospectively analyzed plasma samples (*n* = 80) collected from suspected DILI for HEV markers such as anti-HEV IgM, anti-HEV IgG, and HEV RNA. Anti-HEV antibodies were assessed using commercial ELISA kits. HEV RNA was tested by RT-qPCR targeting HEV ORF2/3, the receiver operating characteristic (ROC) curve was plotted, and a putative threshold for liver function parameters was determined.

**Results:** Out of 80 samples, 12 samples were positive for anti-HEV IgM and anti-HEV IgG, and HEV RNA was detected in seven samples. The median viral load was 3.46 × 10^3^ IU/ml, and the isolated viruses belonged to HEV genotype 1. The level of liver enzymes such as alanine transaminase (ALT) and aspartate transaminase (AST), but not alkaline phosphatase (ALP), was significantly higher in HEV confirmed cases than in non-HEV confirmed cases. We identified a plasma ALT level of at least 415.5 U/L and AST level of at least 332 U/L; ALT/ALP ratio of at least 5.08 could be used as a guide for the patients diagnosed as DILI to be tested for HEV infection. The previous liver function parameters showed high sensitivity and good specificity.

**Conclusion:** Hepatitis E virus was detected in suspected DILI cases. The diagnosis of DILI is not secure until HEV testing is done. Liver function parameters can be used as a guide for HEV testing in suspected DILI cases in countries with limited resources.

## Introduction

Hepatitis E virus (HEV) is a positive-sense, single-stranded RNA virus that causes acute viral hepatitis globally (Rein et al., 2012; Sayed et al., 2015). HEV isolates that cause infections to humans belong to the genus *Orthohepevirus* A, in the *Hepeviridae* family (Smith et al., 2020). There are eight HEV genotypes (HEV 1–8); five of them are associated with human infections (Smith et al., 2016, 2020). HEV-1 and HEV-2 are common in developing countries where the infection is mainly transmitted by the fecal–oral route (World Health Organization [WHO]., 2011; Rein et al., 2012). HEV-3 and HEV-4 are mainly spread by ingestion of undercooked animal products (Masuda et al., 2005; Colson et al., 2010; Huang et al., 2016; El-Mokhtar et al., 2020a; Sayed et al., 2020a,b), and HEV-7 was detected in camels (Lee et al., 2016). HEV-8 was identified in a Bactrian camel, and its role in human infection is still under investigation (Woo et al., 2016).

The HEV genome is about 7.2 kb long, and it includes three open reading frames (ORF 1–3). ORF1 is responsible for viral replication, ORF2 encodes a structural capsid protein, and ORF3 encodes a small phosphoprotein required from viral morphogenesis and release (Ding et al., 2017; Montpellier et al., 2018).

Hepatitis E virus causes acute self-limiting infection in immunocompetent patients, which can progress to acute liver failure (ALF) especially in the elderly (El-Mokhtar et al., 2021; Sayed et al., 2021). Chronic HEV infection is developed in immunocompromised patients such as those with HIV, leukemic individuals, and organ transplant recipients, which can lead to liver cirrhosis (Kamar et al., 2008; Riveiro-Barciela et al., 2014). Besides that, extrahepatic disorders were documented with HEV infections, such as renal, neurological, hematological, and pregnancy-related complications (Pischke et al., 2017; El-Mokhtar and Sayed, 2021). Interferon or ribavirin are used off-label in the treatment of severe and chronic HEV infection (Kamar et al., 2014; Péron et al., 2016).

Drug-induced liver injury (DILI) can lead to ALF, and it affects drug approval, prescription warning, and drug withdrawal from the market (Danan and Benichou, 1993; Fontana et al., 2010). The diagnosis of DILI is difficult and clinically confused with other liver dysfunction causes. DILI is based mainly on the recognition of the likely causative drug and the exclusion of common causes of liver injury, such as viral hepatitis, autoimmune hepatitis, alcoholic hepatitis, and vascular, genetic, and metabolic liver diseases (Fontana et al., 2010). Several studies reported a misdiagnosis of HEV infection in DILI cases in the United States, United Kingdom, Germany, and Scotland (Dalton et al., 2007; Davern et al., 2011; Crossan et al., 2014; Manka et al., 2015). Although there are several scoring systems for DILI such as the Drug-Induced Liver Injury Network and the Roussel Uclaf Causality Assessment Method, neither of them considers HEV diagnosis in the evaluation system (Chen et al., 2012). The actual incidence and registry of DILI is missing in Egypt (Alhaddad et al., 2020). Alhaddad et al. (2020) reported that the prevalence of DILI was 1.38% (75/5452) of all admissions over 1 year in the National Liver Institute. The suspected DILI cases in Egypt are screened for common viral hepatitis such as HCV, HBV, HAV, and CMV, autoimmune hepatitis, but not for HEV. Previous reports recommended that a diagnosis of DILI is not secure without HEV screening, especially in patients with abnormal liver transaminases irrespective of travel history (Crossan et al., 2014; Sayed et al., 2016). The actual prevalence of HEV infection is underestimated in Egypt since the diagnosis of HEV is not routinely enrolled in Egyptian hospitals. Recently, we reported that HEV infection is reported in 10% of acute hepatitis patients of unknown etiology, while DILI cases were excluded from this study (Sayed et al., 2021).

Liver function tests (LFTs), including the assessment of liver transaminases, are simple, inexpensive, and routinely enrolled tests in most hospitals worldwide for screening patients with liver dysfunction. The ratio of alanine aminotransferase (ALT) to alkaline phosphatase (ALP) or *R*-value is used to distinguish between hepatocellular injury and cholestasis (Zimmerman and Ishak, 1995; Yu et al., 2012).

Herein we retrospectively assessed the HEV markers in the plasma of acute hepatitis patients initially diagnosed as DILI. In addition, we aimed to evaluate the possibility of using LFTs as a guide for the clinicians to test the HEV markers in those patients, especially in countries where the diagnosis of HEV is not routinely enrolled.

## Materials and Methods

### Patient Samples

This study included a retrospective analysis of all the available suspected DILI samples (*n* = 80) collected from patients admitted to Assiut University Hospital, Assiut Fever Hospital, AL-Rajhi Liver Hospital, Al-Azhar University Hospital, and Sohag University Hospital, Egypt, from 2016 to 2020. The recruited patients presented with one or more of the acute hepatitis symptoms, such as jaundice, dark urine, pale stool, fever, and abdominal pain. A complete medical history was filled for each patient, and a history of drugs, herbal medicines, and/or over-the-counter medications taken by the patient within the previous 3–6 months was documented. The study design was approved by the Institutional Review Board (IRB Nos. 17200190 and 17300656) at the Faculty of Medicine, Assiut University, Egypt, according to the provisions of the Declaration of Helsinki.

### Diagnosis of Drug-Induced Liver Injury

**FIGURE 1:**
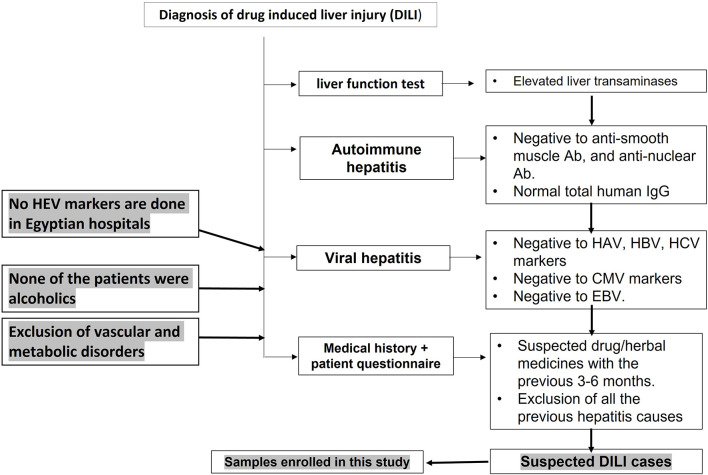
Flow chart showing the diagnosis of drug-induced liver injury in Egyptian hospitals. Acute hepatitis cases were assessed for liver function tests (LFTs), including liver transaminases and bilirubin. Patients with abnormal LFTs were screened for autoimmune hepatitis markers and viral hepatitis markers.

According to EASL guidelines 2019 (European Association for the Study of the Liver., 2019), the diagnosis of DILI was based on the exclusion of other causes of acute liver injury, such as viral hepatitis, autoimmune hepatitis, metabolic disorder, and vascular diseases (Figure 1). Blood samples were screened for LFTs, viral hepatitis markers, and autoimmune markers. LFTs include liver transaminases—ALT and aspartate transaminase (AST)—ALP, and bilirubin. The *R*-value is expressed as [ALT/upper limit of normal (ULN) of ALT]/(ALP/ULN of ALP). Complete blood picture was assessed for eosinophilia. The viral hepatitis tests include screening for HAV, HBV, HCV, CMV, and EBV according to the protocol of Assiut University Hospital and as described previously (Sayed et al., 2021). Briefly, anti-HAV IgM was assessed using a rapid test ELISA assay (CTK Biotech, CA, United States). HBV screening was assessed for HBsAg (ACON Laboratories, Inc., United States), HBV DNA by qPCR, and anti-HBV core IgM (IND Diagnostic, Delta, BC, Canada). Analysis of HCV infection was performed by testing anti-HCV IgG (Atlas Link, United States) and detection of HCV RNA by qPCR. CMV infection was screened using anti-CMV-IgM (MyBioSource, Inc., CA, United States) and PCR for detection of CMV DNA. EBV diagnosis was performed using the monospot test. There is no routine diagnosis for HEV in Egyptian hospitals. The autoimmune hepatitis markers include screening for antinuclear antibodies (ANA), anti-smooth muscle antibodies (ASMA), and total human IgG using ANA Screen IgG ELISA kit (Diagnostic Automation/Cortez Diagnostics Inc., CA, United States), ASMA ELISA Kit (MyBioSource, United States), and (Thermo Fischer Scientific, United States), respectively (Figure 1). Assessment of 24-h urinary copper and serum ceruloplasmin was done. Abdominal and Doppler ultrasound assessments were used to exclude obstructive jaundice and vascular liver diseases, respectively.

### Hepatitis E Virus Serology

Anti-HEV IgM and anti-HEV IgG were assessed in the plasma samples of patients using abia HEV IgM and abia HEV IgG ELISA kits (AB Diagnostic Systems GmbH, Berlin, Germany), respectively, according to the instructions of the manufacturer.

### Molecular Testing for Hepatitis E Virus Nucleic Acid

Total RNA was extracted from plasma samples using QIAamp Viral RNA Mini Kit (Qiagen, Germany), and HEV RNA was assessed using RT-qPCR using targeting HEV ORF2/3 region as described previously (Sayed and Meuleman, 2017; Sayed et al., 2017a,b, 2019). Nested PCR was performed to amplify the 348-bp region of the HEV ORF2, and the amplified region was sequenced to determine the viral genotype (Sayed et al., 2017a,b, 2020a).

### Definition of the Cases

Drug-induced liver injury case refers to any case with clinical presentation of acute liver dysfunction and abnormal liver enzymes and bilirubin. The case tested negative for common viral hepatitis (HAV, HBV, HCV, CMV, and EBV), autoimmune hepatitis markers, and metabolic diseases, and radiological imaging excluded obstructive jaundice and vascular disorders (Figure 1). The medical history and/or a patient questionnaire identified a potential causative drug as suggested in the EASL guidelines (European Association for the Study of the Liver., 2019).

Hepatitis E virus suspected DILI refers to any case which is primarily diagnosed as DILI and, after a reassessment of the samples for HEV markers, and found to be positive to acute HEV markers such as anti-HEV IgM and/or HEV RNA.

Non-HEV suspected DILI refers to any case which is primarily diagnosed as DILI and, after reassessment of the samples for HEV markers, found to be negative to acute HEV markers.

### Statistics

Statistical analyses were done using the GraphPad Prism software 8 (GraphPad Software, La Jolla, United States). The results are presented as median with interquartile range (IQR) unless otherwise specified. *P* < 0.05 was considered significant as determined by two-tailed nonparametric Mann–Whitney test. The receiver operating characteristic (ROC) curve was plotted, and a putative threshold for ALT, *R*-value, and AST was determined. The LF threshold was selected based on the Youden index (sensitivity + specificity − 1), where the threshold with the highest Youden index was selected.

## Results

### Assessment of Hepatitis E Virus Markers in Drug-Induced Liver Injury Samples

**FIGURE 2:**
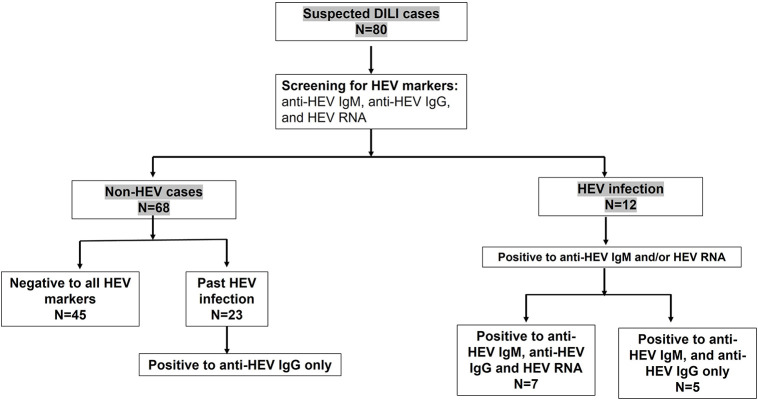
Assessment of hepatitis E virus (HEV) markers in suspected drug-induced liver injury (DILI). Suspected DILI samples (*n* = 80) were reassessed for HEV markers, such as anti-HEV IgM, HEV RNA, and anti-HEV IgG. Twelves samples were positive to anti-HEV IgM and anti-HEV IgG, from which seven samples were also positive to HEV RNA. Forty-five (*n* = 45) samples were negative to all HEV markers, and 23 samples were positive only to anti-HEV IgG, indicating a past infection.

We retrospectively analyzed plasma samples (*n* = 80) previously diagnosed as DILI for HEV markers. The analysis of these samples to HEV markers, such as anti-HEV IgM, anti-HEV IgG, and HEV RNA, revealed that 12 samples (12/80, 15%) were positive to acute hepatitis E (AHE) markers (HEV suspected DILI). These samples were positive to anti-HEV IgM and anti-HEV IgG, and seven samples were also positive to HEV RNA. The median with IQR of HEV load was 3.46 × 10^3^ IU/ml. Sequencing analysis was successful in four cases, and the isolated viruses belonged to HEV genotype 1. While 68 out of 80 samples (85%) were negative to AHE markers (non-HEV suspected DILI), 45 out of 68 samples were negative to all HEV markers and 23 samples were positive only to anti-HEV IgG, suggesting a past HEV infection (Figure 2).

### Demographic and Laboratory Characterization of Hepatitis E Virus Cases

We compared the demographic and laboratory criteria between HEV suspected DILI and non-HEV suspected DILI. The median age was 50 and 43 years, respectively, and there was no significant difference between HEV suspected DILI and non-HEV suspected DILI patients in terms of age and sex (Table 1). The median of ALT (550 U/L) in HEV suspected DILI was significantly higher than in non-HEV suspected DILI (321 U/L), while the ALP and bilirubin levels were comparable in both groups. The *R*-value was significantly higher in HEV suspected DILI compared to non-HEV suspected DILI. The median level of AST was also significantly elevated in the case of an HEV infection (Table 1). Then, we evaluated the medical history for the reported suspected drugs. We found that diclofenac was associated with 17.5% (14/80) of the suspected DILI, five out of 14 (35.7%) were positive to HEV markers, and nine samples (64.3%) tested negative for HEV markers (Table 2). Both ibuprofen and amoxicillin-clavulanic acid were reported in 13.75% (11/80) of the suspected DILI, from which 3/11 (27.3%) and 4/11 (36.4%) were positive for HEV markers, respectively. Acetaminophen, progesterone, acetyl salicylic acid, androgen, thiamazole, atorvastatin, tenoxicam, and carbamazepine were associated with 11.25% (9/80), 8.75% (7/80), 10% (8/80), 7.5% (6/80), 8.75% (7/80), 2.5% (2/80), 5% (4/80), and 1.25% (1/80) of the suspected DILI cases, respectively (Table 2 and Supplementary Table 1). All the previous cases were negative for HEV markers.

**TABLE 1:** Demographic and laboratory criteria and hepatitis E virus (HEV) and non-HEV suspected drug-induced liver injury (DILI).

	**HEV suspected DILI *N* = 12**	**Non-HEV suspected DILI *N* = 68**	**Statistics^a^ S/NS**
Age (years)	50 (40–64)	43 (32–52)	*P* = 0.09, NS
Sex (M/F) Ratio	7/5 1.4:1	38/30 1.26:1	*P* = 0.99, NS
ALT U/L	550 (458–847)	321 (280–394)	*P* < 0.0001, S
ALP U/L	290 (199–350)	314 (239–354)	*P* = 0.46, NS
*R*-value^b^	6.6 (4.3–8.0)	2.96 (2.62–3.97)	*P* < 0.0001, S
AST U/L	462 (348–718)	274 (215–318)	*P* < 0.0001, S
Bilirubin (μmol/L)	143 (115–188)	134 (111–174)	*P* = 0.36, NS
Esinophilia (% positive)	3/12 (25%)	22/68 (32.3%)	*P* = 0.74, NS

*All values are represented as medians and interquartile ranges. ALT: normal range < 40 U/L; AST: normal range < 35 U/L, and ALP: normal range, 46–115 U/L; bilirubin:*

*normal range 1.71 to 20.5 μmol/L.*

*S, significant; NS, non-significant.*

*^*a*^Statistics was calculated using Mann–Whitney test.*

*^*b*^*R*-value = (ALT/ULN)/(ALP/ULN). ULN is the upper limit of normal. The ULN of ALT and ALP was set as 40 and 115 U/L, respectively.*

**TABLE 2:** Drugs reported with HEV and non-HEV suspected DILI cases.

**HEV suspected DILI *N* = 12**	**Non-HEV suspected DILI *N* = 68**
• Ibuprofen (*n* = 3) • Amoxicillin clavulanic acid (*n* = 4) • Diclofenac (*n* = 5)	• Acetaminophen (paracetamol) (*n* = 9) • Ibuprofen (*n* = 8) • Amoxicillin clavulanic acid (*n* = 7) • Diclofenac (*n* = 9) • Progesterone (*n* = 7) • Acetyl salicylic acid (*n* = 8) • Androgen (*n* = 6) • Thiamazole (*n* = 7) • Atorvastatin (*n* = 2) • Tenoxicam (*n* = 4) • Carbamazepine (*n* = 1)

### Liver Function Tests Can Differentiate Hepatitis E Virus Suspected Drug-Induced Liver Injury From Non-Hepatitis E Virus Suspected Drug-Induced Liver Injury

**FIGURE 3:**
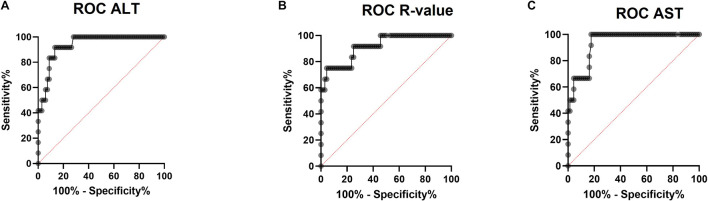
Determination of liver function parameters as a guide for HEV testing using receiver operating characteristic (ROC) curve. The ROC was plotted to identify the plasma ALT **(A)**, R-value **(B)**, and AST **(C)** threshold that differentiates between HEV suspected drug-induced liver injury (DILI) and non-HEV suspected DILI. **(A)** ROC curve for ALT showing the ALT threshold that differentiates between HEV and non-HEV cases. **(B)** ROC curve for *R*-value showing the *R*-value threshold that differentiates between HEV and non-HEV cases. **(C)** ROC curve for AST showing the AST threshold that differentiates between HEV and non-HEV cases.

Then, we performed ROC analysis using the liver function tests to assess if they could be useful to discriminate between HEV suspected DILI from non-HEV suspected DILI. This ROC analysis plots sensitivity *versus* 1 – specificity to assess the overall performance of each test (Figure 3). Using the values of ALT as a cutoff, the area under the ROC curve was 0.93 [95% confidence interval (CI): 0.88–0.99, *P* < 0.0001]. ALT cutoff of at least 415.5 U/L as a trigger for HEV testing would have captured HEV cases with a sensitivity of 91.67% (95% CI: 64.61–99.57%) and has a specificity of 86.76% (95% CI: 76.72–92.88%; positive likelihood ratio, 6.93, *P* < 0.0001; Figure 3A). Using ALT/ALP ratio or *R*-value as a trigger for HEV testing, we found that the area under the ROC curve was 0.91 (95% CI: 0.83–1.00, *P* < 0.0001). An *R*-value of at least 5.08 has a sensitivity of 75% (95% CI: 46.77–91.11%) and a specificity of 95.59% (95% CI: 87.81–98.80%; positive likelihood ratio: 17, *P* < 0.0001; Figure 3B). Using the AST level as a trigger for HEV testing, the area under the ROC curve was 0.94 (95% CI: 0.88–0.99, *P* < 0.0001). An AST level of at least 332 U/L has a sensitivity of 100% (95% CI: 75.75–100.0%) and has a specificity of 82.35% (95% CI: 71.64–89.61%; positive likelihood ratio: 5.67, *P* < 0.0001; Figure 3C). The area under the curve using ALP or bilirubin as a trigger for HEV testing was 0.57 (*p* = 0.45) and 0.58 (*p* = 0.35), respectively.

## Discussion

There are no specific laboratory tests or specific clinical presentations to diagnose DILI, making it more difficult to confirm the diagnosis of DILI. DILI is also a leading cause of acute liver failure that affects drug approval, usage, and/or restriction (Watkins and Seeff, 2006). Several studies have reported that HEV infection is misdiagnosed with DILI cases. Davern et al. (2011) reported that nine out of 318 (3%) of suspected DILI cases in the US were acute HEV infection, and 16% of the patients were positive to anti-HEV IgG. Manka et al. (2015) reported that eight out of 80 (10%) of ALF cases in German hospitals were caused by HEV, and half of the cases were initially misdiagnosed as idiosyncratic DILI. Dalton et al. (2007) likewise reported that 21% of patients who were initially misdiagnosed as criterion-referenced DILI were autochthonous HEV, and 22% of autochthonous HEV-infected patients were incorrectly labeled as DILI before reassessment with HEV markers. In a parallel line, HEV was recorded in four out of 80 cases of ALF in the Scottish liver transplant unit; three of them were initially erroneously ascribed to DILI (Crossan et al., 2014). Therefore, HEV testing should be considered for all DILI causality, especially when the more common etiologies have been excluded. In most developed countries, HEV diagnosis becomes routinely enrolled in acute hepatitis cases—therefore, the risk of misdiagnosis between HEV infection and DILI is low—while HEV diagnosis is still underestimated in most developing countries—therefore, the percentage of HEV cases that are misdiagnosed as DILI cases is expected to be high.

In this study, we retrospectively analyzed plasma samples initially labeled as DILI for HEV markers. We found that 15% of the samples were indeed recent or ongoing AHE infection. Sequencing analysis revealed that the isolated viruses belong to HEV genotype 1 as described in our previous cohorts (El-Mokhtar et al., 2020b, 2021; Sayed et al., 2021). While 85% of samples were negative to AHE markers, 33.8% of samples were positive only to anti-HEV IgG, indicating a past HEV infection. We and previous studies have reported that HEV-1 is circulating in Egypt, and the anti-HEV IgG seroprevalence is high among Egyptians, especially in rural communities. The prevalence could range from 10 to 84% in pregnant women in the Egyptian villages (Darwish et al., 1996; Stoszek et al., 2006a,b; Delarocque-Astagneau et al., 2012; Sayed et al., 2021). The difference between the seroprevalence in this study and in previous studies could be attributed to the geographic distribution, the analyzed subjects, ELISA kit used in the analysis, time/year of analysis, *etc*.

In this study, we compared the demographic characteristics and LFTs of the DILI cases with HEV cases. We did not find a difference between the two groups in terms of age, gender, and presence of eosinophilia. However, the level of ALT, AST, and ALT/ALP ratio or *R*-value was significantly elevated in the setting of HEV infections. Similarly, Wallace et al. (2017) reported that the level of ALT and ALT/ALP ratio could differentiate between acute HEV infection and DILI. Compared to confirmed DILI cases, Dalton et al. (2007) reported that HEV-infected patients had significantly higher serum ALT, ALT/ALP ratio, and lower serum bilirubin.

In this study, we aimed to identify the best LFTs that could be a guide for HEV screening. Our results showed that the ALT cutoff of 415.5 U/L, AST cutoff of 332 U/L, and *R*-value threshold of 5.08 as a trigger for HEV testing has a sensitivity of 91.67, 100, and 75%, respectively, and a specificity of 86.76, 82, and 95.59%, respectively, to differentiate between HEV-infected cases and non-HEV infected cases, while ALP and/or bilirubin were not good candidates to discriminate between DILI groups in terms of HEV infection. It is worthy to note that the LFT threshold values in this study reflect the test results of this cohort and could differ at different places depending on the methodology procedure and instrumentation, reference values of LFTs, patient criteria, reported suspected drugs, *etc*. Similar to our results, Wallace et al. (2017) reported that ALT ≥ 300 had a sensitivity of 98.6% and specificity of 30.3% to discriminate between HEV infection (*n* = 74) and non-viral causes of liver dysfunction, such as criterion-referenced DILI (*n* = 69), patients with common bile ducts stones (*n* = 87), and patients with other causes of hepatitis, such as DILI and decompensated chronic liver disease (*n* = 530). In the previous report, the authors also reported that an ALT/ALP threshold of 2 had a sensitivity of 100% and a specificity of 9.4% to differentiate between HEV group and other groups (Wallace et al., 2017). In a parallel line, Harvala et al. (2014) reported that HEV screening should be limited to patients with ALT ≥ 100 U/L. The previous finding was concluded from the observation that 100% (25/25) of HEV IgM-positive patients has ALT level ≥ 100 U/L and 92% (23/25) of HEV IgM-positive patients has ALT level ≥ 300 U/L (Harvala et al., 2014). Interestingly, similar to our observation, Wallace et al. (2017) showed that bilirubin and ALP were not good parameters to discriminate between HEV infection and other liver dysfunction causes, including DILI.

The use of LF parameters as a guide for HEV testing was performed in terms of HEV genotype 3 infections, and both reports were conducted on patients from developed countries (the United Kingdom and Scotland) (Harvala et al., 2014; Wallace et al., 2017). Herein we showed the use of LFTs as a guide for HEV testing in the setting of HEV genotype 1 infection, and the patients enrolled in this study were from developing countries such as Egypt.

One limitation of this study is that the number of samples is not big (*n* = 80). The absence of liver biopsy samples to confirm DILI, due to the retrospective nature of the study, is also a limitation. Further studies including a larger number of patients are needed to ascertain our findings, and performing multi-center studies on different HEV genotypes is also warranted. Moreover, the difference in clinical outcome between cases of HEV and DILI was not assessed in this study, and this could be further evaluated in future prospective studies.

Clinicians can benefit from the LFT parameters to predict the possibility of HEV infection in misdiagnosed DILI cases. This step could reduce the complications associated with HEV infections, especially for the high-risk group, by taking appropriate curative measures.

In conclusion, it is difficult to distinguish between DILI and HEV infections depending on the clinical presentation without testing for HEV. Our results recommend using the LFTs as a guide to screen for acute HEV infection in suspected DILI since they show high sensitivity and acceptable specificity, especially in countries where the diagnosis of HEV infection is not routinely enrolled. ALT level of at least 415.5 U/L, AST at least 332 U/L, or *R*-value of at least 5.08 could categorize the patients recently diagnosed as DILI to be tested for HEV infection.

## Data Availability Statement

The original contributions presented in the study are included in the article/Supplementary Material, further inquiries can be directed to the corresponding authors.

## Ethics Statement

The studies involving human participants were reviewed and approved by the Institutional Review Board (IRB Nos. 17200190 and 17300656) at the Faculty of Medicine, Assiut University, Egypt, according to the provisions of the Declaration of Helsinki. The ethics committee waived the requirement of written informed consent for participation.

## Author Contributions

ME-M, MT, AA, MM, and IS: design the study. HR, MT, AA, MF, MS, and EE: resources. ME-M, HR, MT, AA, MF, MS, EE, AAE, MM, and IS: methodology and analysis. HR and IS: supervision. IS: write the manuscript. All authors reviewed and edited the manuscript, contributed to the article, and approved the submitted version.

## Conflict of Interest

The authors declare that the research was conducted in the absence of any commercial or financial relationships that could be construed as a potential conflict of interest.

## Publisher’s Note

All claims expressed in this article are solely those of the authors and do not necessarily represent those of their affiliated organizations, or those of the publisher, the editors and the reviewers. Any product that may be evaluated in this article, or claim that may be made by its manufacturer, is not guaranteed or endorsed by the publisher.
