# Supplementary material for: The Unmet Needs of Hepatitis E Virus Diagnosis in Suspected Drug-Induced Liver Injury in Limited Resource Setting
Source: Front Microbiol. 2021 Oct 8;12:737486. doi: 10.3389/fmicb.2021.737486 (PMC8533821; doi:10.3389/fmicb.2021.737486)
Supplement: Supplementary file 1 [file Table_1.DOCX]

**Supple Table (1): Time interval between drug intake and symptoms appeared**

| **Drug** | **Time interval between drug and symptoms (Days)** |
| --- | --- |
| Ibuprofen | 16.55± 7.216 |
| Amoxicillin Clavulanic acid | 8.909± 2.982 |
| Diclofenac | 17.86±5.112 |
| Acetaminophen | 13.44 ± 4.902 |
| Progesterone | 28.29 ± 5.187 |
| Acetyl Salicylic acid | 16.25± 3.370 |
| Androgen | 25.83 ± 7.055 |
| Thiamazole | 32.17± 17.43 |
| Atorvastatin | 32.00± 2.828 |
| Tenoxicam | 12.00± 2.944 |
| Carbamazepine | 44.00±0.00 |

All values are expressed as mean ± SD
